# Incorporation of *in situ* generated 3,3′-(sulfane­diyl)bis­(1-methyl-1,3-imidazolidine-2-thione) into a one-dimensional Cu^I^ coordination polymer with sulfur-bridged {Cu^I^_4_S_10_}_*n*_ central cores

**DOI:** 10.1107/S2056989022004911

**Published:** 2022-05-17

**Authors:** Tarlok Singh Lobana, Ray J. Butcher, Jerry P. Jasinski

**Affiliations:** a Department of Chemistry, Guru Nanak Dev University, Amritsar 143 005, India; b Department of Chemistry, Howard University, 525 College Street NW, Washington DC 20059, USA; c Department of Chemistry, Keene State College, Keene NH 03435-2001, USA; University of Aberdeen, Scotland

**Keywords:** crystal structure, copper(I) thione complex, one-dimensional coordination polymer

## Abstract

The reaction of [Cu(CH_3_CN)_4_](BF_4_) with 1-methyl-1,3-imidazolidine-2-thione {SC_3_H_4_(NMe)NH}, under aerobic conditions at room temperature, yielded an unusual one-dimensional coordination polymer, namely, *catena*-poly[[[(1-meth­yl-1,3-imidazolidine-2-thione-κ*S*)copper(I)]-μ-(1-methyl-1,3-imidazolidine-2-thione)-κ^2^ *S*:*S*-copper(I)-μ-[3,3′-(sulfanedi­yl)bis­(1-methyl-1,3-imidazolidine-2-thione)]-κ^5^ *S*,*S*′,*S*′′:*S*,*S*′′] bis­(tetra­fluorido­borate)], {[Cu_2_(C_4_H_8_N_2_S)_2_(C_8_H_14_N_4_S_3_)](BF_4_)_2_}_*n*_ or [Cu_4_(κ^5^:*L* ^1^—N—S—N—*L* ^1^)_2_(κ^1^:*L* ^1^—NH)_2_(κ^2^:*L* ^1^—NH)_2_]_*n*_(BF_4_)_4*n*_ **1** [*L* ^1^ = SC_3_ H_4_(NMe)NH] with sulfur-bridged {Cu^I^_4_S_10_}_*n*_ central cores. The *in situ* generated bis­(1-methyl-1,3-imidazolidinyl-2-thione) sulfide [{SC_3_H_4_(NMe)NSN(NMe)C_3_H_4_S; abbrev. *L* ^1^—N—S—N—*L* ^1^] ligand, in combin­ation with 1-methyl-1,3-imidazolidine-2-thione (*L* ^1^—NH) ligands, construct this coordination polymer. Each Cu^I^ ion is bonded to four sulfur donor atoms in a distorted tetra­hedral geometry and the formation of this polymer solely by sulfur donor atoms with {Cu^I^_4_S_10_}_*n*_ central cores, is the first such example in copper–heterocyclic-2-thione chemistry.

## Abstract 2

The reaction of [Cu(CH_3_CN)_4_](BF_4_) with 1-methyl-1,3-imidazolidine-2-thione {SC_3_H_4_(NMe) NH} forms the one-dimensional coordination polymer [Cu_4_(κ^5^:*L* ^1^—N—S—N—*L* ^1^)_2_(κ^1^:*L* ^1^—NH)_2_(κ^2^: *L* ^1^—NH)_2_]_*n*_(BF_4_)_4n_ {*L* ^1^ = SC_3_H_4_(NMe)NH} with sulfur-bridged {Cu^I^_4_S_10_}_*n*_ central cores.

## 1. Chemical context

**Figure 1:**
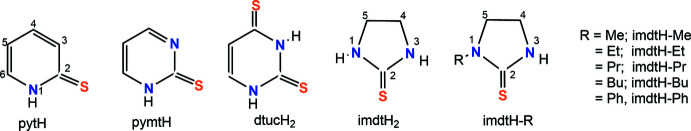
A selected list of heterocyclic-2-thio­nes.

The coordination chemistry of the coinage metals (Cu–Au) with heterocyclic-2-thione ligands (Fig. 1 ▸) is of considerable inter­est as these metals exhibit a wide range of coordination geometries, giving rise to coordination compounds of differing nuclearity, namely, mononuclear, homo- and hetero-bridged di-nuclear, clusters and coordination polymers (Lobana, 2021 ▸; Raper, 1994 ▸, 1996 ▸, 1997 ▸; García-Vázquez *et al.*, 1999 ▸). It has been noted that coordination compounds of these metals have displayed promising bio-activity and, in addition, several copper-based reactions are involved in the activation of C=S (thione) bonds (Lobana, 2021 ▸). 
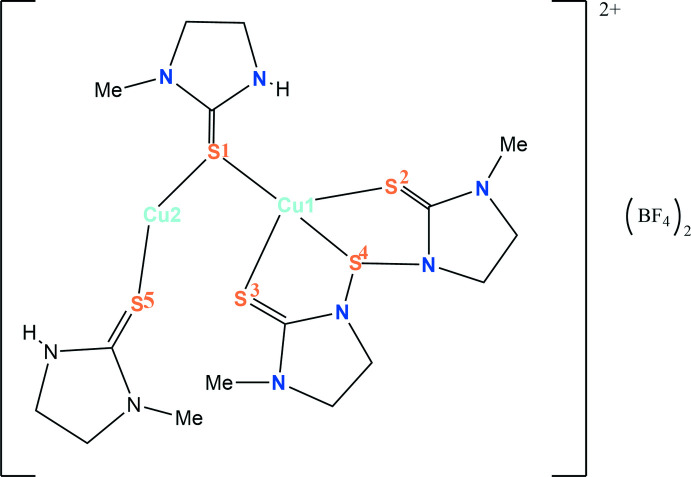


As part of out ongoing studies in this area, we now describe the synthesis and structure of the title coordination polymer, **1**.

## 2. Structural commentary

**Figure 2:**
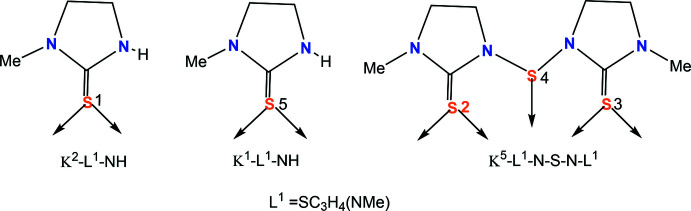
Bonding pattern of 1-methyl-1,3-imidazodine-2-thione and its *in situ* generated bis­(1-methyl-1,3-imidazolidinyl-2-thione)sulfide.

The analytical data of the colourless crystals (see *Synthesis and crystallization*) correspond to the empirical composition C_16_H_30_B_2_Cu_2_F_8_N_8_S_5_ and its crystal structure revealed the formation of an unusual coordination polymer, {Cu_4_(κ^5^:*L* ^1^—N—S—N—*L* ^1^)_2_(κ^1^:*L* ^1^—NH)_2_(κ^2^:*L* ^1^—NH)_2_}_*n*_^−^(BF_4_)_4n_(**1**) [*L* ^1^= SC_3_H_4_(NMe)]. There is *in situ* generation of a new thio-ligand, namely, bis­(1-methyl-1,3-imidazolidinyl-2-thione) sulfide [SC_3_H_4_(NMe)N—S—NSC_3_H_4_(NMe); abbrev. *L* ^1^—N—S—N—*L* ^1^] in which a sulfur atom connects two —NH groups of two imidazolidine rings. Fig. 2 ▸ shows the bonding patterns of *L* ^1^—NH, and the new thio-ligand, in the polymer **1**.

**Figure 3:**
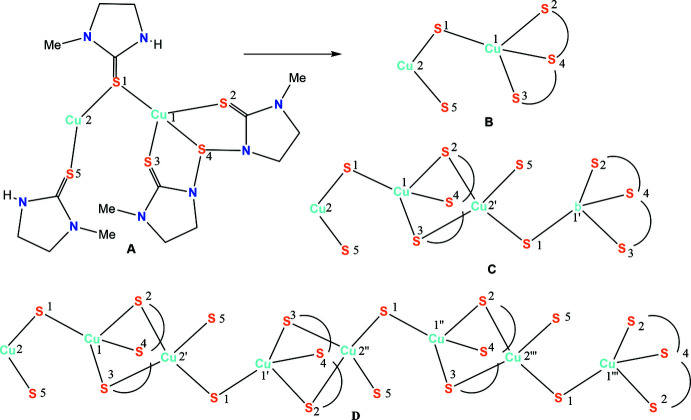
The basic repeating unit, **A**; the basic repeating unit with imidazolidine rings omitted, **B**; the tetra­nuclear unit, **C**; a part of the polymer, **D**.

**Figure 4:**
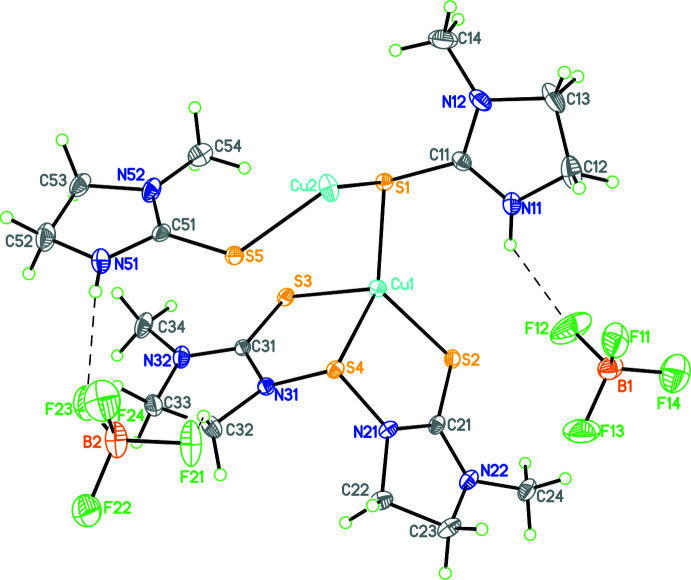
The contents of the asymmetric unit. N—H⋯F hydrogen bonds are shown as dashed lines. Atomic displacement parameters are drawn at the 30% probability level.

**Figure 6:**
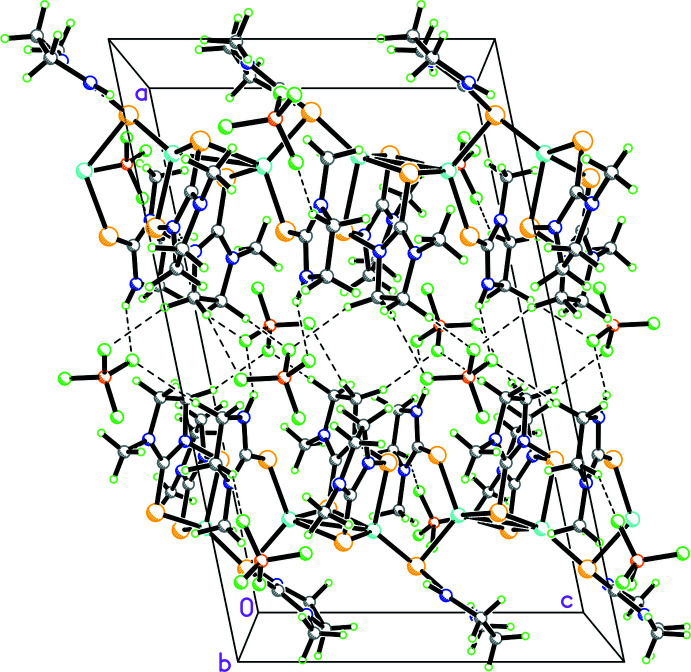
Packing viewed along the *b-*axis direction. N—H⋯F hydrogen bonds and C—H⋯F inter­actions shown as dashed lines.

The construction of the polymer **1** is believed to occur as represented in Fig. 3 ▸. Here the basic repeat unit is **A**, which is shown in a simplified way as unit **B** (omitting the imidazolidine rings). Two such **B** units combine to form a tetra­nuclear unit **C**, a basic building block, to construct the polymer **1**. The building block **C** exhibits all three patterns of ligand bonding as represented in Fig. 2 ▸. The crystals of the polymer are monoclinic in the space group *P*2_1_/*c*. Geometric parameters are given in Table 1 ▸ Fig. 4 ▸ shows the basic dinuclear unit, in which there are three bonding patterns: bridging bidentate sulfur (κ^2^-*L* ^1^—NH), monodentate sulfur (κ^1^-*L* ^1^—NH), and *in situ* generated penta­dentate sulfur ligand (κ^5^-*L* ^1^—N—S—N—*L* ^1^) (Fig. 2 ▸). The combining of two dinuclear moieties gives rise to a tetra­nuclear moiety as shown in Figs. 2 ▸ and 5 ▸. The chains of the polymer are hydrogen bonded to BF_4_ ions lying between the chains by multiple weak C—H⋯F inter­actions as shown in Fig. 6 ▸ and listed in Table 2 ▸.

Cu1 is bonded to four sulfur donor atoms (S1–S4) (Table 1 ▸). Here the thione (C=S) sulfur donor atoms are more strongly bonded relative to the sulfur atom of the —N—S—N— moiety. The Cu2—S2 and Cu2—S3 bond distances are the longest, while the other two Cu2—S1 and Cu2—S5 distances are short, and comparable to the Cu1—sulfur (S1–S3) bond distances, as noted above. The Cu⋯Cu separation of 2.9074 (8) Å, does not reveal any metal–metal inter­action (the sum of the van der Waals radii of the Cu atoms is 2.80 Å; Huheey *et al.*, 1993 ▸). The C—S bond distances fall in the range 1.699 (4) to 1.723 (4) Å, and lie between a typical double- and single-bond distance (C=S ≃ 1.68 Å; C—S ≃ 1.81 Å; Huheey *et al.*, 1993 ▸). Finally, the geometry about Cu1 is significantly distorted from a regular tetra­hedron, as revealed by the S—Cu1—S bond angles, which fall in the range 89.72 (4) to 131.86 (4)° and this is illustrated by the τ_4_’ parameter of 0.725 (Okuniewski *et al.*, 2015 ▸); in comparison, the geometry of Cu2 is less distorted, with S—Cu2—S bond angles in the range 100.47 (4)–122.65 (4)° and a τ_4_’ parameter of 0.842.

**Figure 7:**
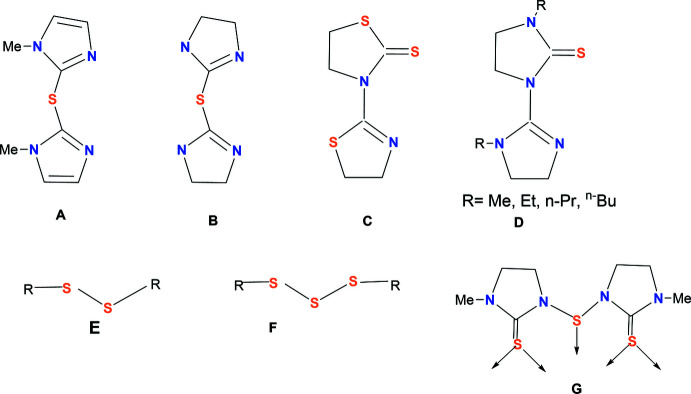
*In situ* generated thio-ligands, **A**–**G** in heterocyclic-2-thione chemistry.

The *in situ* formation of the new thio-ligand appears in line with the metal-mediated variable chemical activity of N,S-donor thio-ligands, such as the activation of C=S (thione) bonds (Lobana, 2021 ▸; Lobana *et al.*, 2010 ▸), as well as the activation of C—H and N—H bonds (Lobana *et al.*, 2012 ▸, 2007 ▸, 2008 ▸). The oxidation of heterocyclic-2-thio­nes such as benzo-1,3-thia­zoline-2-thione, pyridine-2-thione, pyrimidine-2-thione, 1,3-imidazolidine-2-thione, quinoline-2-thione, 1,3,4-thia­diazole-2,5-di­thia­zone and benzo-1,3-thia­zoline-2-thione to their di­sulfides/tris­ulfides, followed by coordination to the metal ions, has been reported previously (Raper, 1994 ▸; Lobana, 2021 ▸). In the present case, in relation to the activation of C=S (thione) bonds, *in situ* generated thio-ligands, **A–F**, have been reported (Lobana, 2021 ▸; Raper, 1994 ▸; Ferrari *et al.*, 1981 ▸; Kadooka *et al.*, 1976 ▸; Simmons *et al.*, 1979 ▸ *;* Jeannin *et al.*, 1979 ▸) (Fig. 7 ▸). In the **E** and **F** ligands, *R* is 2-pyridyl-, 2-pyrimidyl-, etc. and these have C—(S)_*n*_—C (*n* = 2, 3) groups, connecting the heterocyclic rings. In the ligand **G**, there is one N—S—N connecting group, two thione groups, and thus it is a new and different ligand.

## 3. Supra­molecular features

The BF_4_^−^ anions lying between the chains are involved in inter­actions with various N—H and C—H hydrogen atoms of the thio-ligands (Figs. 4 ▸ and 6 ▸). Consider the dimeric unit shown in Fig. 4 ▸. Here the N11—H hydrogen atom inter­acts with the F12 and F12*A* fluorine atoms of one BF_4_^−^ anion while the N51—H hydrogen atom inter­acts with the F23 fluorine atom of the second BF_4_^−^ ion. Various other F atoms of both BF_4_^−^ ions accept C—H⋯F inter­actions from the imidazolidine ring and the N-methyl group. The distances and angles involving hydrogen-bond inter­actions are shown in Table 2 ▸. In summary, the distances and angles are given as follows: N⋯F = 2.74 (2)–2.764 (11) Å, H⋯F = 2.12–2.15 Å and N—H⋯F = 124–129°; C⋯F = 2.93 (2)–3.57 (2)Å; H⋯F = 2.09–2.64 Å; C—H⋯F = 111–170°. The N⋯F distances are less than the sum of van der Waals radii of N and F, namely, 3.05 to 3.15 Å, and likewise the C⋯F distances are either less than or comparable to the sum of van der Waals radii of C and F, namely, 3.15 to 3.30 Å (Huheey *et al.*, 1993 ▸).

## 4. Database survey

In the light of the novelty of thio-ligands under discussion, a few examples of coordination compounds of pyridine-2-thione, pyrimidine-2-thione, di­thio­uracil and 1,3-imidazolidine-2-thio­nes, are delineated here (Fig. 1 ▸). For example, pyridine-2-thione (pytH) in combination with copper(I) halides has formed a variety of coordination compounds: namely, mononuclear [Cu*X*(κ^1^ *S*-pytH)(PPh_3_)_2_] (*X* = Cl, Br), dinuclear, [Cu_2_Br_2_(μ-*S*-pytH)_2_(PPh_3_)_2_], [Cu_2_Br_2_(μ-*P*, P-dppe)_2_(κ^1^ *S*-pytH)_2_] (dppe = Ph_2_P-CH_2_-CH_2_-PPh_2_), [Cu_2_(μ-*S*-pytH)_2_(κ^1^ *S*-pytH)_4_]*X*_2_ (*X* = Cl, Br), [Cu_2_I_2_(μ-pytH)_2_(κ^1^ *S*-pytH)_2_] and trinuclear, [Cu_3_I_3_(μ-*P*,*P*-dppe)_3_(κ^1^ *S*-pytH)] (Lobana *et al.*, 1989 ▸, 2002 ▸; Karagiannidis *et al.*, 1989 ▸; Cox *et al.*, 2000 ▸; Stergioudis *et al.*, 1987 ▸; Mentzafos *et al.*, 1989 ▸; Davies *et al.*, 1997 ▸; Lobana *et al.*, 2003 ▸, 2005 ▸).

The examples of coordination polymers include a hexa­nuclear linear polymer, {Cu_6_(μ_3_-*S*-pytH)_4_(μ-*S*-pytH)_2_(I_4_)(μ-*I*)_2_-}_*n*_·2*n*CH_3_CN, pyrimidine-2-thione (pymtH) and 2,4-di­thio­uracil (dtucH_2_) based linear Cu^I^ chain polymers, [Cu(μ-N,S-pymtH)*X*]_*n*_ (*X* = Cl, Br),{Cu(μ-*S*,*S*-dtucH_2_)(PPh_3_)*X*}_*n*_ (*X* = Cl, Br, I), imidazolidine-2-thione (imdtH_2_) based polymers, [{Cu_6_(μ_3_-*S*-imdtH_2_)_2_(μ-*S*-imdtH_2_)_4_ *X*_2_(μ-*X*)_4_}_*n*_] (*X* = Cl, Br, I-halogen bridged), {Cu_6_(μ_3_-*S*-imdtH_2_)_4_(μ-imdtH_2_)_2_(μ-*I*)_2_I_4_}_*n*_ (sulfur-bridged), and an octa­nuclear polymer, {Cu_8_(μ_3_-*S*-imdtH_2_)_4_(μ-*S*-imdtH_2_)_4_(κ^1^-*Cl*)_8_}_*n*_. *N*-Phenyl-1,3-imidazolidine-2-thione also forms a linear chain polymer, {Cu_3_I_3_(imdtH-Ph)_3_}_*n*_, with alternate Cu_2_I_2_ and Cu_2_S_2_ dimeric units forming the chains (Lobana *et al.*, 2003 ▸, 2005 ▸, 2006 ▸, 2009 ▸; Li *et al.*, 2005 ▸; Sultana *et al.*, 2010 ▸; Aulakh *et al.*, 2017 ▸).

In the literature, there are limited reports of complexes with ionic copper(I) salts, and the reported mono-, or di-nuclear ionic complexes have BF_4_^−^, ClO_4_^−^, PF_6_^−^ *etc*., outside the metal coordination sphere (Lobana, 2021 ▸). The present study provides a basic background to develop a new class of polymers using copper(I) ionic salts with heterocyclic-2-thio­nes. The resulting polymeric materials with a central metal atom linked only to sulfur donor atoms may have inter­esting conductivity properties.

## 5. Synthesis and crystallization

All solvents were of HPLC grade and were stored over mol­ecular sieves. The precursor, tetra­kis­(aceto­nitrile)­copper(I) tetra­fluoro­borate, [Cu(CH_3_CN)_4_](BF_4_), was prepared by the slow addition of HBF_4_ acid (from boric acid H_3_BO_3_ + HF acid in a plastic beaker) to a solution of Cu_2_O (0.200 g; 1.4 mmol) in dry aceto­nitrile (25 ml) in a round-bottom flask. The mixture slowly became colourless and a white salt settled in the flask. The mother liquor was removed and the salt was extracted with diethyl ether, followed by evaporation, which gave solid [Cu(CH_3_CN)_4_](BF_4_).

**Synthesis of 1-methyl-1,3-imidazolidine-2-thione**

Carbon di­sulfide (4.1 ml, 76 mmol) was added to a cooled solution of 1-methyl-ethyl­enedi­amine (CH_3_-NH-CH_2_-CH_2_-NH_2_) dissolved in ethanol (10 ml) followed by the addition of 10 ml of water (García-Vázquez *et al.*, 2005 ▸). A white precipitate formed, and the contents were heated at 333 K, followed by the further addition of CS_2_. The precipitate initially dissolved, but shortly thereafter, a large amount of precipitate was deposited. The reaction mixture was heated under reflux for 1h, followed by the addition of conc. HCl (0.5 mL). It was further refluxed for one h, and placed for cooling, and precipitate formed was filtered and washed with cold acetone. Colour: white. Yield: 1.15 g, 50%; m.p. 351–354 K.

**Synthesis of 1**

To a solution of [Cu(CH_3_CN)_4_](BF_4_) (0.050 g, 0.15 mmol) in methanol (10 mL) was added a solution of the thio-ligand, SC_3_H_4_(NMe)NH (0.036 g, 0.31 mmol) in methanol. The mixture was stirred for about half an hour, giving rise to the formation of a clear pale-yellow solution. It was kept undisturbed for evaporation at room temperature. The colour of the solution turned green and a colourless crystalline compound was formed at the bottom, which was separated and dried at room temperature. Yield: 0.025 g; 40%; m.p. 450–452 K. Analysis found: C 24.52; H 3.69; N 13.87; S 20.50; C_16_H_30_B_2_Cu_2_F_8_N_8_S_5_ requires: C 24.14; H 3.77; N 14.08; S 20.11%.

## 6. Refinement

Crystal data, data collection and structure refinement details are summarized in Table 3 ▸. All hydrogen atoms were placed geometrically and refined as riding atoms with *U*_iso_(H) = 1.2U_eq_(C) or 1.5*U*_eq_(methyl C). Both BF_4_ anions and one imidazoline ring are disordered over two sets of sites with occupancy ratios of 0.66 (2)/0.34 (2), 0.72 (2)/0.28 (2), and 0.622 (6)/0.378 (6), respectively.

## Supplementary Material

Crystal structure: contains datablock(s) I. DOI: 10.1107/S2056989022004911/hb8015sup1.cif

Structure factors: contains datablock(s) I. DOI: 10.1107/S2056989022004911/hb8015Isup2.hkl

CCDC reference: 2150195

Additional supporting information:  crystallographic information; 3D view; checkCIF report

## Figures and Tables

**Figure 5:**
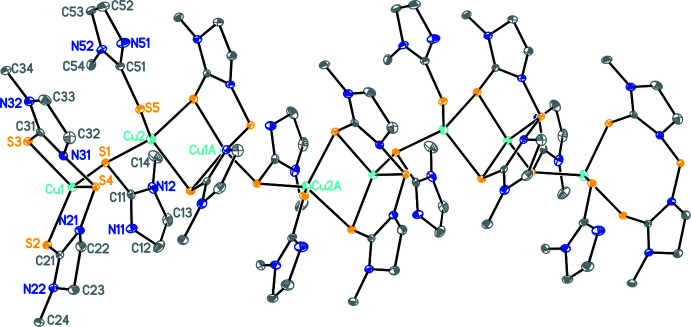
The tetra­nuclear repeating unit. H atoms and BF_4_^−^ anions are omitted for clarity.

**Table 1:** Selected geometric parameters (Å, °)

Cu1—S1	2.2590 (10)	Cu2—S5	2.2696 (11)
Cu1—S2	2.2997 (10)	Cu2—S1	2.3179 (11)
Cu1—S3	2.3423 (10)	Cu2—S3^ii^	2.4364 (11)
Cu1—S4	2.4162 (10)	Cu2—S2^ii^	2.5338 (11)
Cu1—Cu2^i^	2.9074 (8)		
			
S1—Cu1—S2	131.86 (4)	S5—Cu2—S1	122.65 (4)
S1—Cu1—S3	105.12 (4)	S5—Cu2—S3^ii^	109.15 (4)
S2—Cu1—S3	110.79 (4)	S1—Cu2—S3^ii^	102.24 (4)
S1—Cu1—S4	120.66 (4)	S5—Cu2—S2^ii^	105.31 (4)
S2—Cu1—S4	90.89 (4)	S1—Cu2—S2^ii^	114.68 (4)
S3—Cu1—S4	89.72 (4)	S3^ii^—Cu2—S2^ii^	100.47 (4)

Symmetry codes: (i) 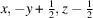 ; (ii) 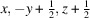 .

**Table 2:** Hydrogen-bond geometry (Å, °)

*D*—H⋯*A*	*D*—H	H⋯*A*	*D*⋯*A*	*D*—H⋯*A*
N11—H11*A*⋯F12	0.88	2.12	2.764 (11)	129
N11—H11*A*⋯F12*A*	0.88	2.15	2.74 (2)	124
N51—H51*A*⋯F23	0.88	2.20	2.995 (12)	150
C13—H13*B*⋯F12^iii^	0.99	2.63	3.329 (17)	128
C13—H13*B*⋯F12*A* ^iii^	0.99	2.59	3.22 (3)	122
C14—H14*B*⋯F14*A* ^iii^	0.98	2.59	3.331 (14)	133
C14—H14*C*⋯F11*A* ^iv^	0.98	2.64	3.119 (17)	111
C22—H22*B*⋯F24^v^	0.99	2.63	3.293 (8)	125
C22—H22*B*⋯F24*A* ^v^	0.99	2.61	3.395 (17)	136
C23—H23*A*⋯F24^v^	0.99	2.56	3.164 (8)	119
C23—H23*B*⋯F22^vi^	0.99	2.63	3.453 (8)	140
C24—H24*C*⋯F13	0.98	2.58	3.321 (11)	133
C32—H32*A*⋯F21	0.99	2.33	3.259 (15)	155
C32—H32*A*⋯F21*A*	0.99	2.46	3.38 (3)	155
C32—H32*A*⋯F23*A*	0.99	2.61	3.287 (16)	126
C32—H32*B*⋯F24^i^	0.99	2.56	3.499 (9)	158
C32—H32*B*⋯F24*A* ^i^	0.99	2.59	3.569 (18)	170
C33—H33*A*⋯F21^i^	0.99	2.33	3.182 (12)	144
C33—H33*A*⋯F21*A* ^i^	0.99	2.31	3.16 (3)	145
C34—H34*A*⋯F13*A* ^i^	0.98	2.62	3.154 (18)	114
C34—H34*B*⋯F22^vii^	0.98	2.60	3.247 (8)	123
C53—H53*B*⋯F13^viii^	0.99	2.36	3.269 (16)	152
C54—H54*A*⋯S1	0.98	2.89	3.835 (8)	162
C54—H54*C*⋯F11^i^	0.98	2.39	3.335 (10)	162
C53*A*—H53*D*⋯F22^vii^	0.99	2.52	3.49 (3)	167
C54*A*—H54*D*⋯F23	0.98	2.09	2.933 (16)	144
C54*A*—H54*D*⋯F23*A*	0.98	2.60	3.43 (2)	143
C54*A*—H54*E*⋯F23*A* ^vii^	0.98	2.47	3.36 (2)	152

Symmetry codes: (i) 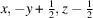 ; (iii) 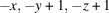 ; (iv) 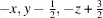 ; (v) 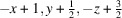 ; (vi) 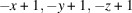 ; (vii) 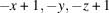 ; (viii) 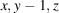 .

**Table 3:** Experimental details

Crystal data	
Chemical formula	[Cu_2_(C_4_H_8_N_2_S)_2_(C_8_H_14_N_4_S_3_)](BF_4_)_2_
*M*_r_	795.48
Crystal system, space group	Monoclinic, *P*2_1_/*c*
Temperature (K)	100
*a*, *b*, *c* (Å)	19.0636 (7), 13.6989 (3), 11.5770 (4)
β (°)	101.734 (3)
*V* (Å^3^)	2960.16 (17)
*Z*	4
Radiation type	Mo *K*α
μ (mm^−1^)	1.87
Crystal size (mm)	0.32 × 0.24 × 0.16

Data collection	
Diffractometer	Xcalibur, Eos, Gemini
Absorption correction	Multi-scan (*CrysAlis PRO*; Rigaku, 2019 ▸)
*T*_min_, *T*_max_	0.673, 1.000
No. of measured, independent and observed [*I* > 2σ(*I*)] reflections	33195, 8277, 7139
*R*_int_	0.032
(sin θ/λ)_max_ (Å^−1^)	0.694

Refinement	
*R*[*F* ^2^ > 2σ(*F* ^2^)], *wR*(*F* ^2^), *S*	0.055, 0.138, 1.09
No. of reflections	8277
No. of parameters	506
No. of restraints	497
H-atom treatment	H-atom parameters constrained
Δρ_max_, Δρ_min_ (e Å^−3^)	1.91, −1.29

Computer programs: *CrysAlis PRO* and *CrysAlis RED* (Rigaku, 2019 ▸), *SHELXT* (Sheldrick, 2015*a* ▸), *SHELXL2018/3* (Sheldrick, 2015*b* ▸) and *SHELXTL* (Sheldrick, 2008 ▸).

## supplementary crystallographic information

### Crystal data

**Table:**

[Cu_2_(C_4_H_8_N_2_S)_2_(C_8_H_14_N_4_S_3_)](BF_4_)_2_	*F*(000) = 1608
*M**_r_* = 795.48	*D*_x_ = 1.785 Mg m^−^^3^
Monoclinic, *P*2_1_/*c*	Mo *K*α radiation, λ = 0.71073 Å
*a* = 19.0636 (7) Å	Cell parameters from 11503 reflections
*b* = 13.6989 (3) Å	θ = 3.1–32.3°
*c* = 11.5770 (4) Å	µ = 1.87 mm^−^^1^
β = 101.734 (3)°	*T* = 100 K
*V* = 2960.16 (17) Å^3^	Chunk, colorless
*Z* = 4	0.32 × 0.24 × 0.16 mm

### Data collection

**Table:**

Xcalibur, Eos, Gemini diffractometer	7139 reflections with *I* > 2σ(*I*)
Detector resolution: 16.1500 pixels mm^-1^	*R*_int_ = 0.032
ω scans	θ_max_ = 29.6°, θ_min_ = 3.1°
Absorption correction: multi-scan (CrysAlisPro; Rigaku, 2019)	*h* = −26→26
*T*_min_ = 0.673, *T*_max_ = 1.000	*k* = −17→19
33195 measured reflections	*l* = −16→16
8277 independent reflections	

### Refinement

**Table:**

Refinement on *F*^2^	Primary atom site location: structure-invariant direct methods
Least-squares matrix: full	Secondary atom site location: difference Fourier map
*R*[*F*^2^ > 2σ(*F*^2^)] = 0.055	Hydrogen site location: inferred from neighbouring sites
*wR*(*F*^2^) = 0.138	H-atom parameters constrained
*S* = 1.09	*w* = 1/[σ^2^(*F*_o_^2^) + (0.039*P*)^2^ + 14.326*P*] where *P* = (*F*_o_^2^ + 2*F*_c_^2^)/3
8277 reflections	(Δ/σ)_max_ < 0.001
506 parameters	Δρ_max_ = 1.91 e Å^−^^3^
497 restraints	Δρ_min_ = −1.29 e Å^−^^3^

### Special details

**Table:**

Geometry. All esds (except the esd in the dihedral angle between two l.s. planes) are estimated using the full covariance matrix. The cell esds are taken into account individually in the estimation of esds in distances, angles and torsion angles; correlations between esds in cell parameters are only used when they are defined by crystal symmetry. An approximate (isotropic) treatment of cell esds is used for estimating esds involving l.s. planes.
Refinement. Refined as a 2-component twin.

### Fractional atomic coordinates and isotropic or equivalent isotropic displacement parameters (Å^2^)

**Table:**

	*x*	*y*	*z*	*U*_iso_*/*U*_eq_	Occ. (<1)
Cu1	0.17200 (3)	0.29294 (3)	0.41072 (4)	0.02467 (12)	
Cu2	0.19437 (3)	0.17473 (4)	0.67264 (5)	0.03398 (14)	
S1	0.10220 (5)	0.21261 (7)	0.51531 (8)	0.02188 (18)	
C11	0.05713 (19)	0.2930 (3)	0.5887 (3)	0.0239 (7)	
N11	0.0615 (2)	0.3899 (3)	0.5829 (4)	0.0330 (8)	
H11A	0.083532	0.421452	0.534404	0.040*	
C12	0.0247 (3)	0.4373 (4)	0.6680 (5)	0.0490 (13)	
H12A	−0.006637	0.491000	0.630846	0.059*	
H12B	0.059362	0.462956	0.736785	0.059*	
C13	−0.0185 (3)	0.3537 (4)	0.7028 (5)	0.0476 (13)	
H13A	−0.014559	0.351379	0.789336	0.057*	
H13B	−0.069603	0.359160	0.663905	0.057*	
N12	0.01487 (19)	0.2670 (3)	0.6605 (3)	0.0339 (8)	
C14	−0.0110 (3)	0.1701 (4)	0.6768 (6)	0.0540 (15)	
H14A	0.021727	0.121783	0.654344	0.081*	
H14B	−0.058844	0.161726	0.627217	0.081*	
H14C	−0.013555	0.160793	0.759755	0.081*	
S2	0.16050 (5)	0.44336 (7)	0.32065 (8)	0.02344 (18)	
C21	0.2452 (2)	0.4870 (3)	0.3691 (3)	0.0220 (7)	
N21	0.30019 (17)	0.4367 (2)	0.4393 (3)	0.0230 (6)	
C22	0.3681 (2)	0.4915 (3)	0.4478 (4)	0.0295 (8)	
H22A	0.396466	0.466287	0.391463	0.035*	
H22B	0.397611	0.489061	0.528730	0.035*	
C23	0.3406 (2)	0.5937 (3)	0.4154 (4)	0.0335 (9)	
H23A	0.339174	0.633096	0.486565	0.040*	
H23B	0.370330	0.627624	0.367087	0.040*	
N22	0.26779 (19)	0.5744 (2)	0.3468 (3)	0.0265 (7)	
C24	0.2230 (3)	0.6540 (3)	0.2916 (4)	0.0328 (9)	
H24A	0.183100	0.627598	0.232845	0.049*	
H24B	0.251451	0.698081	0.252849	0.049*	
H24C	0.204067	0.689934	0.351782	0.049*	
S3	0.20669 (5)	0.17479 (7)	0.28720 (9)	0.02339 (18)	
C31	0.29663 (18)	0.1774 (3)	0.3396 (3)	0.0196 (6)	
N31	0.33350 (16)	0.2506 (2)	0.4087 (3)	0.0208 (6)	
C32	0.4110 (2)	0.2266 (4)	0.4393 (4)	0.0351 (9)	
H32A	0.427437	0.218752	0.525530	0.042*	
H32B	0.440017	0.277800	0.410917	0.042*	
C33	0.4152 (2)	0.1304 (3)	0.3749 (4)	0.0353 (10)	
H33A	0.445475	0.137149	0.315261	0.042*	
H33B	0.435200	0.078091	0.431056	0.042*	
N32	0.34084 (19)	0.1094 (3)	0.3188 (3)	0.0296 (7)	
C34	0.3211 (3)	0.0212 (3)	0.2520 (4)	0.0391 (11)	
H34A	0.272500	0.027972	0.204794	0.059*	
H34B	0.322777	−0.034105	0.306143	0.059*	
H34C	0.354725	0.009827	0.199567	0.059*	
S4	0.29655 (5)	0.32490 (6)	0.49486 (8)	0.02048 (17)	
S5	0.30419 (5)	0.12584 (7)	0.64829 (8)	0.02327 (18)	
C51	0.3161 (4)	0.0135 (4)	0.5964 (6)	0.0248 (12)	0.622 (6)
N51	0.3829 (4)	−0.0246 (5)	0.6150 (7)	0.0386 (14)	0.622 (6)
H51A	0.421277	0.005232	0.654537	0.046*	0.622 (6)
C52	0.3839 (4)	−0.1209 (5)	0.5618 (8)	0.0435 (16)	0.622 (6)
H52A	0.406344	−0.169866	0.620714	0.052*	0.622 (6)
H52B	0.409746	−0.119777	0.495817	0.052*	0.622 (6)
C53	0.3053 (6)	−0.1418 (8)	0.5183 (14)	0.0364 (17)	0.622 (6)
H53A	0.295008	−0.158421	0.433318	0.044*	0.622 (6)
H53B	0.289407	−0.196357	0.562912	0.044*	0.622 (6)
N52	0.2695 (3)	−0.0493 (4)	0.5398 (5)	0.0325 (12)	0.622 (6)
C54	0.1936 (4)	−0.0347 (6)	0.4976 (7)	0.0364 (16)	0.622 (6)
H54A	0.181113	0.032894	0.512284	0.055*	0.622 (6)
H54B	0.166873	−0.079161	0.539145	0.055*	0.622 (6)
H54C	0.181205	−0.048104	0.412704	0.055*	0.622 (6)
C51A	0.2840 (6)	0.0129 (8)	0.5822 (11)	0.0267 (18)	0.378 (6)
N51A	0.2168 (6)	−0.0181 (7)	0.5374 (10)	0.0333 (19)	0.378 (6)
H51B	0.177838	0.012996	0.546084	0.040*	0.378 (6)
C52A	0.2167 (7)	−0.1106 (8)	0.4719 (12)	0.041 (2)	0.378 (6)
H52C	0.200468	−0.100562	0.385886	0.049*	0.378 (6)
H52D	0.185884	−0.160175	0.498967	0.049*	0.378 (6)
C53A	0.2945 (9)	−0.1396 (13)	0.502 (3)	0.038 (2)	0.378 (6)
H53C	0.301324	−0.198020	0.553806	0.046*	0.378 (6)
H53D	0.313059	−0.153626	0.430208	0.046*	0.378 (6)
N52A	0.3305 (5)	−0.0534 (7)	0.5651 (9)	0.0344 (16)	0.378 (6)
C54A	0.4056 (6)	−0.0555 (11)	0.6164 (15)	0.044 (3)	0.378 (6)
H54D	0.423838	0.011507	0.627346	0.066*	0.378 (6)
H54E	0.431180	−0.090733	0.563981	0.066*	0.378 (6)
H54F	0.413072	−0.088521	0.693007	0.066*	0.378 (6)
B1	0.1527 (3)	0.6462 (4)	0.6039 (5)	0.0388 (12)	
B2	0.5451 (3)	0.1715 (4)	0.7345 (5)	0.0456 (14)	
F11	0.1661 (5)	0.6294 (6)	0.7267 (5)	0.0572 (16)	0.662 (12)
F12	0.1231 (8)	0.5666 (8)	0.5451 (12)	0.094 (3)	0.662 (12)
F13	0.2172 (5)	0.6656 (7)	0.5761 (9)	0.0743 (18)	0.662 (12)
F14	0.1088 (5)	0.7262 (5)	0.5796 (7)	0.0744 (18)	0.662 (12)
F11A	0.1480 (10)	0.6571 (13)	0.7155 (12)	0.062 (2)	0.338 (12)
F12A	0.1085 (16)	0.5744 (17)	0.549 (3)	0.098 (5)	0.338 (12)
F13A	0.2223 (8)	0.6300 (14)	0.5893 (19)	0.076 (3)	0.338 (12)
F14A	0.1344 (9)	0.7331 (9)	0.5380 (14)	0.078 (2)	0.338 (12)
F21	0.4996 (7)	0.2511 (8)	0.7097 (12)	0.065 (2)	0.699 (14)
F22	0.6131 (3)	0.1982 (5)	0.7198 (8)	0.0571 (14)	0.699 (14)
F23	0.5171 (4)	0.0954 (5)	0.6579 (7)	0.0553 (15)	0.699 (14)
F24	0.5484 (5)	0.1432 (6)	0.8493 (5)	0.0664 (16)	0.699 (14)
F21A	0.5052 (15)	0.2535 (16)	0.718 (3)	0.064 (4)	0.301 (14)
F22A	0.6182 (7)	0.1936 (13)	0.7683 (16)	0.063 (2)	0.301 (14)
F23A	0.5376 (9)	0.1170 (12)	0.6304 (11)	0.056 (2)	0.301 (14)
F24A	0.5289 (10)	0.1148 (13)	0.8249 (14)	0.065 (2)	0.301 (14)

### Atomic displacement parameters (Å^2^)

**Table:**

	*U*^11^	*U*^22^	*U*^33^	*U*^12^	*U*^13^	*U*^23^
Cu1	0.0257 (2)	0.0236 (2)	0.0267 (2)	0.00048 (17)	0.01001 (18)	0.00126 (18)
Cu2	0.0295 (3)	0.0422 (3)	0.0289 (3)	0.0125 (2)	0.0030 (2)	−0.0037 (2)
S1	0.0228 (4)	0.0213 (4)	0.0222 (4)	−0.0001 (3)	0.0059 (3)	0.0007 (3)
C11	0.0178 (16)	0.0312 (19)	0.0220 (17)	0.0025 (14)	0.0022 (13)	0.0023 (14)
N11	0.0318 (18)	0.0260 (17)	0.043 (2)	0.0039 (14)	0.0112 (16)	−0.0041 (15)
C12	0.044 (3)	0.052 (3)	0.048 (3)	0.014 (2)	0.005 (2)	−0.020 (3)
C13	0.043 (3)	0.068 (4)	0.037 (2)	0.025 (3)	0.019 (2)	0.004 (2)
N12	0.0274 (17)	0.047 (2)	0.0311 (18)	0.0093 (16)	0.0150 (14)	0.0086 (16)
C14	0.039 (3)	0.059 (3)	0.069 (4)	0.000 (2)	0.024 (3)	0.028 (3)
S2	0.0247 (4)	0.0210 (4)	0.0253 (4)	0.0036 (3)	0.0067 (3)	0.0002 (3)
C21	0.0309 (19)	0.0174 (15)	0.0181 (15)	0.0016 (13)	0.0060 (13)	−0.0008 (13)
N21	0.0261 (15)	0.0173 (13)	0.0251 (15)	−0.0037 (11)	0.0043 (12)	0.0003 (12)
C22	0.030 (2)	0.0273 (19)	0.030 (2)	−0.0064 (15)	0.0027 (16)	0.0010 (16)
C23	0.041 (2)	0.0226 (18)	0.035 (2)	−0.0088 (17)	0.0040 (18)	−0.0011 (16)
N22	0.0377 (19)	0.0178 (14)	0.0245 (16)	−0.0016 (13)	0.0071 (14)	−0.0009 (12)
C24	0.048 (3)	0.0207 (18)	0.031 (2)	0.0043 (17)	0.0121 (19)	0.0037 (15)
S3	0.0260 (4)	0.0188 (4)	0.0248 (4)	−0.0012 (3)	0.0036 (3)	−0.0024 (3)
C31	0.0221 (16)	0.0192 (15)	0.0183 (15)	0.0023 (12)	0.0059 (13)	0.0053 (12)
N31	0.0217 (14)	0.0207 (14)	0.0205 (14)	0.0014 (11)	0.0053 (11)	0.0020 (11)
C32	0.0212 (19)	0.044 (2)	0.040 (2)	0.0056 (17)	0.0049 (17)	0.001 (2)
C33	0.031 (2)	0.034 (2)	0.044 (2)	0.0103 (17)	0.0145 (19)	0.0108 (19)
N32	0.0371 (19)	0.0249 (16)	0.0289 (17)	0.0091 (14)	0.0119 (14)	0.0014 (13)
C34	0.063 (3)	0.0231 (19)	0.035 (2)	0.0112 (19)	0.019 (2)	−0.0022 (17)
S4	0.0255 (4)	0.0190 (4)	0.0172 (4)	−0.0005 (3)	0.0049 (3)	0.0012 (3)
S5	0.0251 (4)	0.0208 (4)	0.0252 (4)	−0.0006 (3)	0.0082 (3)	−0.0019 (3)
C51	0.037 (3)	0.018 (2)	0.024 (3)	0.002 (2)	0.018 (3)	0.003 (2)
N51	0.039 (3)	0.027 (3)	0.054 (3)	0.008 (2)	0.018 (3)	−0.004 (3)
C52	0.055 (3)	0.030 (3)	0.048 (3)	0.013 (3)	0.017 (3)	−0.003 (3)
C53	0.059 (4)	0.020 (3)	0.034 (4)	0.003 (3)	0.020 (3)	−0.003 (3)
N52	0.048 (3)	0.019 (2)	0.032 (2)	0.005 (2)	0.011 (2)	−0.0053 (19)
C54	0.048 (4)	0.034 (4)	0.026 (4)	−0.006 (3)	0.005 (3)	−0.004 (3)
C51A	0.039 (4)	0.019 (3)	0.026 (3)	0.001 (3)	0.016 (4)	0.004 (3)
N51A	0.044 (4)	0.024 (4)	0.032 (4)	−0.002 (3)	0.007 (4)	−0.003 (3)
C52A	0.057 (4)	0.028 (4)	0.038 (4)	−0.005 (4)	0.008 (4)	−0.006 (3)
C53A	0.058 (4)	0.023 (4)	0.037 (5)	0.002 (3)	0.018 (4)	−0.004 (4)
N52A	0.046 (3)	0.024 (3)	0.038 (3)	0.004 (3)	0.019 (3)	−0.002 (3)
C54A	0.045 (5)	0.031 (6)	0.061 (7)	0.011 (5)	0.022 (5)	0.001 (6)
B1	0.048 (3)	0.035 (3)	0.035 (3)	−0.009 (2)	0.012 (2)	−0.008 (2)
B2	0.048 (3)	0.047 (3)	0.038 (3)	0.020 (3)	0.000 (2)	−0.008 (2)
F11	0.088 (5)	0.055 (4)	0.036 (2)	0.014 (3)	0.028 (3)	−0.001 (2)
F12	0.156 (8)	0.055 (4)	0.076 (4)	−0.055 (5)	0.031 (5)	−0.030 (3)
F13	0.086 (3)	0.069 (5)	0.080 (4)	−0.019 (3)	0.047 (3)	0.007 (4)
F14	0.092 (4)	0.062 (3)	0.069 (3)	0.012 (3)	0.018 (3)	0.005 (3)
F11A	0.089 (5)	0.057 (4)	0.048 (3)	0.009 (4)	0.029 (3)	−0.005 (3)
F12A	0.136 (10)	0.067 (8)	0.085 (8)	−0.051 (8)	0.008 (8)	−0.020 (7)
F13A	0.082 (5)	0.074 (6)	0.084 (5)	−0.010 (5)	0.048 (4)	0.015 (6)
F14A	0.096 (4)	0.063 (4)	0.077 (4)	−0.004 (4)	0.024 (3)	0.009 (4)
F21	0.074 (4)	0.061 (4)	0.048 (4)	0.041 (3)	−0.012 (3)	−0.016 (3)
F22	0.061 (3)	0.055 (2)	0.059 (3)	0.008 (2)	0.020 (3)	−0.004 (3)
F23	0.056 (3)	0.048 (3)	0.060 (3)	0.007 (2)	0.007 (2)	−0.015 (2)
F24	0.074 (3)	0.072 (4)	0.048 (3)	0.007 (3)	0.000 (3)	0.000 (3)
F21A	0.071 (7)	0.059 (7)	0.052 (8)	0.038 (7)	−0.010 (7)	−0.018 (7)
F22A	0.066 (4)	0.065 (4)	0.054 (4)	0.012 (3)	0.003 (4)	−0.007 (4)
F23A	0.061 (4)	0.050 (4)	0.056 (4)	0.011 (3)	0.009 (3)	−0.011 (3)
F24A	0.070 (4)	0.063 (4)	0.058 (4)	0.006 (3)	0.004 (3)	−0.003 (3)

### Geometric parameters (Å, º)

**Table:**

Cu1—S1	2.2590 (10)	C34—H34C	0.9800
Cu1—S2	2.2997 (10)	S5—C51	1.684 (6)
Cu1—S3	2.3423 (10)	S5—C51A	1.735 (10)
Cu1—S4	2.4162 (10)	C51—N52	1.312 (8)
Cu1—Cu2^i^	2.9074 (8)	C51—N51	1.352 (8)
Cu2—S5	2.2696 (11)	N51—C52	1.458 (8)
Cu2—S1	2.3179 (11)	N51—H51A	0.8800
Cu2—S3^ii^	2.4364 (11)	C52—C53	1.507 (11)
Cu2—S2^ii^	2.5338 (11)	C52—H52A	0.9900
S1—C11	1.723 (4)	C52—H52B	0.9900
C11—N12	1.319 (5)	C53—N52	1.484 (9)
C11—N11	1.333 (5)	C53—H53A	0.9900
N11—C12	1.471 (6)	C53—H53B	0.9900
N11—H11A	0.8800	N52—C54	1.445 (9)
C12—C13	1.512 (8)	C54—H54A	0.9800
C12—H12A	0.9900	C54—H54B	0.9800
C12—H12B	0.9900	C54—H54C	0.9800
C13—N12	1.477 (6)	C51A—N52A	1.312 (11)
C13—H13A	0.9900	C51A—N51A	1.348 (12)
C13—H13B	0.9900	N51A—C52A	1.477 (11)
N12—C14	1.442 (6)	N51A—H51B	0.8800
C14—H14A	0.9800	C52A—C53A	1.505 (14)
C14—H14B	0.9800	C52A—H52C	0.9900
C14—H14C	0.9800	C52A—H52D	0.9900
S2—C21	1.706 (4)	C53A—N52A	1.481 (12)
C21—N22	1.317 (5)	C53A—H53C	0.9900
C21—N21	1.373 (5)	C53A—H53D	0.9900
N21—C22	1.482 (5)	N52A—C54A	1.434 (12)
N21—S4	1.669 (3)	C54A—H54D	0.9800
C22—C23	1.516 (6)	C54A—H54E	0.9800
C22—H22A	0.9900	C54A—H54F	0.9800
C22—H22B	0.9900	B1—F11A	1.323 (12)
C23—N22	1.475 (5)	B1—F12	1.347 (9)
C23—H23A	0.9900	B1—F13	1.358 (9)
C23—H23B	0.9900	B1—F12A	1.365 (13)
N22—C24	1.450 (5)	B1—F14	1.374 (8)
C24—H24A	0.9800	B1—F13A	1.387 (13)
C24—H24B	0.9800	B1—F11	1.411 (8)
C24—H24C	0.9800	B1—F14A	1.419 (12)
S3—C31	1.699 (4)	B2—F21A	1.349 (13)
C31—N32	1.312 (5)	B2—F24	1.374 (8)
C31—N31	1.381 (5)	B2—F21	1.387 (8)
N31—C32	1.484 (5)	B2—F24A	1.388 (13)
N31—S4	1.678 (3)	B2—F22	1.390 (8)
C32—C33	1.524 (6)	B2—F23A	1.400 (12)
C32—H32A	0.9900	B2—F22A	1.400 (13)
C32—H32B	0.9900	B2—F23	1.403 (8)
C33—N32	1.464 (6)	F11—F11A	0.512 (15)
C33—H33A	0.9900	F13—F13A	0.514 (17)
C33—H33B	0.9900	F14—F14A	0.758 (14)
N32—C34	1.442 (5)	F22—F22A	0.554 (15)
C34—H34A	0.9800	F23—F23A	0.626 (15)
C34—H34B	0.9800	F24—F24A	0.571 (15)
			
S1—Cu1—S2	131.86 (4)	C51—N51—H51A	124.0
S1—Cu1—S3	105.12 (4)	C52—N51—H51A	124.0
S2—Cu1—S3	110.79 (4)	N51—C52—C53	102.5 (6)
S1—Cu1—S4	120.66 (4)	N51—C52—H52A	111.3
S2—Cu1—S4	90.89 (4)	C53—C52—H52A	111.3
S3—Cu1—S4	89.72 (4)	N51—C52—H52B	111.3
S1—Cu1—Cu2^i^	142.97 (3)	C53—C52—H52B	111.3
S2—Cu1—Cu2^i^	56.80 (3)	H52A—C52—H52B	109.2
S3—Cu1—Cu2^i^	54.01 (3)	N52—C53—C52	103.9 (6)
S4—Cu1—Cu2^i^	91.89 (3)	N52—C53—H53A	111.0
S5—Cu2—S1	122.65 (4)	C52—C53—H53A	111.0
S5—Cu2—S3^ii^	109.15 (4)	N52—C53—H53B	111.0
S1—Cu2—S3^ii^	102.24 (4)	C52—C53—H53B	111.0
S5—Cu2—S2^ii^	105.31 (4)	H53A—C53—H53B	109.0
S1—Cu2—S2^ii^	114.68 (4)	C51—N52—C54	127.5 (6)
S3^ii^—Cu2—S2^ii^	100.47 (4)	C51—N52—C53	110.9 (6)
S5—Cu2—Cu1^ii^	118.66 (3)	C54—N52—C53	121.6 (6)
S1—Cu2—Cu1^ii^	118.48 (3)	N52—C54—H54A	109.5
S3^ii^—Cu2—Cu1^ii^	51.07 (3)	N52—C54—H54B	109.5
S2^ii^—Cu2—Cu1^ii^	49.42 (3)	H54A—C54—H54B	109.5
C11—S1—Cu1	111.11 (13)	N52—C54—H54C	109.5
C11—S1—Cu2	97.47 (13)	H54A—C54—H54C	109.5
Cu1—S1—Cu2	95.46 (4)	H54B—C54—H54C	109.5
N12—C11—N11	110.7 (4)	N52A—C51A—N51A	110.0 (9)
N12—C11—S1	124.6 (3)	N52A—C51A—S5	126.0 (8)
N11—C11—S1	124.7 (3)	N51A—C51A—S5	124.0 (8)
C11—N11—C12	111.3 (4)	C51A—N51A—C52A	111.7 (9)
C11—N11—H11A	124.4	C51A—N51A—H51B	124.2
C12—N11—H11A	124.4	C52A—N51A—H51B	124.2
N11—C12—C13	101.8 (4)	N51A—C52A—C53A	102.0 (9)
N11—C12—H12A	111.4	N51A—C52A—H52C	111.4
C13—C12—H12A	111.4	C53A—C52A—H52C	111.4
N11—C12—H12B	111.4	N51A—C52A—H52D	111.4
C13—C12—H12B	111.4	C53A—C52A—H52D	111.4
H12A—C12—H12B	109.3	H52C—C52A—H52D	109.2
N12—C13—C12	103.1 (4)	N52A—C53A—C52A	103.9 (9)
N12—C13—H13A	111.1	N52A—C53A—H53C	111.0
C12—C13—H13A	111.1	C52A—C53A—H53C	111.0
N12—C13—H13B	111.1	N52A—C53A—H53D	111.0
C12—C13—H13B	111.1	C52A—C53A—H53D	111.0
H13A—C13—H13B	109.1	H53C—C53A—H53D	109.0
C11—N12—C14	126.9 (4)	C51A—N52A—C54A	126.8 (10)
C11—N12—C13	110.5 (4)	C51A—N52A—C53A	111.4 (9)
C14—N12—C13	121.0 (4)	C54A—N52A—C53A	120.8 (10)
N12—C14—H14A	109.5	N52A—C54A—H54D	109.5
N12—C14—H14B	109.5	N52A—C54A—H54E	109.5
H14A—C14—H14B	109.5	H54D—C54A—H54E	109.5
N12—C14—H14C	109.5	N52A—C54A—H54F	109.5
H14A—C14—H14C	109.5	H54D—C54A—H54F	109.5
H14B—C14—H14C	109.5	H54E—C54A—H54F	109.5
C21—S2—Cu1	99.67 (12)	F11A—B1—F12	118.7 (12)
C21—S2—Cu2^i^	95.24 (13)	F11A—B1—F13	117.0 (11)
Cu1—S2—Cu2^i^	73.78 (3)	F12—B1—F13	110.2 (8)
N22—C21—N21	109.4 (3)	F11A—B1—F12A	112.1 (13)
N22—C21—S2	125.4 (3)	F12—B1—F12A	13 (2)
N21—C21—S2	125.2 (3)	F13—B1—F12A	122.3 (16)
C21—N21—C22	109.7 (3)	F11A—B1—F14	87.1 (8)
C21—N21—S4	127.0 (3)	F12—B1—F14	111.5 (9)
C22—N21—S4	122.9 (3)	F13—B1—F14	109.8 (6)
N21—C22—C23	101.3 (3)	F12A—B1—F14	100.8 (15)
N21—C22—H22A	111.5	F11A—B1—F13A	113.2 (11)
C23—C22—H22A	111.5	F12—B1—F13A	97.0 (14)
N21—C22—H22B	111.5	F13—B1—F13A	21.6 (7)
C23—C22—H22B	111.5	F12A—B1—F13A	110.1 (13)
H22A—C22—H22B	109.3	F14—B1—F13A	131.3 (8)
N22—C23—C22	102.1 (3)	F11A—B1—F11	21.3 (7)
N22—C23—H23A	111.4	F12—B1—F11	110.4 (8)
C22—C23—H23A	111.4	F13—B1—F11	106.5 (7)
N22—C23—H23B	111.4	F12A—B1—F11	108.5 (15)
C22—C23—H23B	111.4	F14—B1—F11	108.3 (5)
H23A—C23—H23B	109.2	F13A—B1—F11	96.6 (10)
C21—N22—C24	125.7 (4)	F11A—B1—F14A	111.8 (9)
C21—N22—C23	111.5 (3)	F12—B1—F14A	112.1 (11)
C24—N22—C23	120.6 (3)	F13—B1—F14A	81.0 (8)
N22—C24—H24A	109.5	F12A—B1—F14A	107.1 (13)
N22—C24—H24B	109.5	F14—B1—F14A	31.4 (6)
H24A—C24—H24B	109.5	F13A—B1—F14A	101.9 (8)
N22—C24—H24C	109.5	F11—B1—F14A	130.7 (8)
H24A—C24—H24C	109.5	F21A—B2—F24	106.4 (15)
H24B—C24—H24C	109.5	F21A—B2—F21	5 (2)
C31—S3—Cu1	99.03 (13)	F24—B2—F21	109.0 (7)
C31—S3—Cu2^i^	98.92 (12)	F21A—B2—F24A	111.4 (14)
Cu1—S3—Cu2^i^	74.92 (3)	F24—B2—F24A	23.9 (6)
N32—C31—N31	110.6 (3)	F21—B2—F24A	111.7 (12)
N32—C31—S3	124.0 (3)	F21A—B2—F22	106.2 (16)
N31—C31—S3	125.4 (3)	F24—B2—F22	109.4 (5)
C31—N31—C32	110.0 (3)	F21—B2—F22	109.2 (8)
C31—N31—S4	124.0 (3)	F24A—B2—F22	126.6 (8)
C32—N31—S4	120.7 (3)	F21A—B2—F23A	111.6 (13)
N31—C32—C33	103.0 (3)	F24—B2—F23A	131.2 (9)
N31—C32—H32A	111.2	F21—B2—F23A	106.9 (11)
C33—C32—H32A	111.2	F24A—B2—F23A	110.6 (9)
N31—C32—H32B	111.2	F22—B2—F23A	88.2 (8)
C33—C32—H32B	111.2	F21A—B2—F22A	111.1 (14)
H32A—C32—H32B	109.1	F24—B2—F22A	86.8 (8)
N32—C33—C32	104.1 (3)	F21—B2—F22A	115.7 (12)
N32—C33—H33A	110.9	F24A—B2—F22A	105.7 (9)
C32—C33—H33A	110.9	F22—B2—F22A	22.9 (6)
N32—C33—H33B	110.9	F23A—B2—F22A	106.1 (9)
C32—C33—H33B	110.9	F21A—B2—F23	113.6 (15)
H33A—C33—H33B	109.0	F24—B2—F23	109.8 (6)
C31—N32—C34	125.9 (4)	F21—B2—F23	108.1 (7)
C31—N32—C33	112.3 (3)	F24A—B2—F23	86.9 (8)
C34—N32—C33	121.8 (4)	F22—B2—F23	111.3 (5)
N32—C34—H34A	109.5	F23A—B2—F23	25.8 (6)
N32—C34—H34B	109.5	F22A—B2—F23	124.5 (9)
H34A—C34—H34B	109.5	F11A—F11—B1	69.6 (17)
N32—C34—H34C	109.5	F13A—F13—B1	82 (2)
H34A—C34—H34C	109.5	F14A—F14—B1	77.5 (11)
H34B—C34—H34C	109.5	F11—F11A—B1	89.1 (19)
N21—S4—N31	105.82 (16)	F13—F13A—B1	76.0 (19)
N21—S4—Cu1	97.21 (12)	F14—F14A—B1	71.0 (10)
N31—S4—Cu1	98.47 (11)	F22A—F22—B2	79.6 (16)
C51—S5—Cu2	120.6 (2)	F23A—F23—B2	76.8 (13)
C51A—S5—Cu2	101.0 (3)	F24A—F24—B2	79.4 (15)
N52—C51—N51	110.3 (6)	F22—F22A—B2	77.5 (16)
N52—C51—S5	130.6 (5)	F23—F23A—B2	77.3 (13)
N51—C51—S5	119.1 (5)	F24—F24A—B2	76.7 (16)
C51—N51—C52	112.0 (6)		
			
Cu1—S1—C11—N12	−177.0 (3)	N51A—C51A—N52A—C53A	0 (2)
Cu2—S1—C11—N12	−78.2 (4)	S5—C51A—N52A—C53A	−177.9 (16)
Cu1—S1—C11—N11	1.3 (4)	C52A—C53A—N52A—C51A	6 (2)
Cu2—S1—C11—N11	100.1 (4)	C52A—C53A—N52A—C54A	175.4 (15)
N12—C11—N11—C12	6.4 (5)	F12—B1—F11—F11A	−117 (3)
S1—C11—N11—C12	−172.1 (3)	F13—B1—F11—F11A	123 (3)
C11—N11—C12—C13	−14.2 (5)	F12A—B1—F11—F11A	−104 (3)
N11—C12—C13—N12	15.6 (5)	F14—B1—F11—F11A	5 (3)
N11—C11—N12—C14	170.6 (5)	F13A—B1—F11—F11A	143 (3)
S1—C11—N12—C14	−10.9 (7)	F14A—B1—F11—F11A	31 (4)
N11—C11—N12—C13	4.8 (5)	F11A—B1—F13—F13A	85 (4)
S1—C11—N12—C13	−176.6 (3)	F12—B1—F13—F13A	−55 (4)
C12—C13—N12—C11	−13.4 (5)	F12A—B1—F13—F13A	−60 (4)
C12—C13—N12—C14	179.9 (5)	F14—B1—F13—F13A	−178 (3)
Cu1—S2—C21—N22	−177.1 (3)	F11—B1—F13—F13A	65 (4)
Cu2^i^—S2—C21—N22	108.5 (3)	F14A—B1—F13—F13A	−165 (4)
Cu1—S2—C21—N21	1.6 (3)	F11A—B1—F14—F14A	142.8 (17)
Cu2^i^—S2—C21—N21	−72.8 (3)	F12—B1—F14—F14A	−97.4 (17)
N22—C21—N21—C22	−9.8 (4)	F13—B1—F14—F14A	25.0 (17)
S2—C21—N21—C22	171.3 (3)	F12A—B1—F14—F14A	−105.2 (19)
N22—C21—N21—S4	177.5 (3)	F13A—B1—F14—F14A	24 (2)
S2—C21—N21—S4	−1.5 (5)	F11—B1—F14—F14A	141.0 (15)
C21—N21—C22—C23	21.3 (4)	F12—B1—F11A—F11	72 (3)
S4—N21—C22—C23	−165.6 (3)	F13—B1—F11A—F11	−64 (3)
N21—C22—C23—N22	−23.3 (4)	F12A—B1—F11A—F11	84 (3)
N21—C21—N22—C24	−169.8 (4)	F14—B1—F11A—F11	−175 (3)
S2—C21—N22—C24	9.2 (6)	F13A—B1—F11A—F11	−41 (3)
N21—C21—N22—C23	−7.0 (5)	F14A—B1—F11A—F11	−155 (3)
S2—C21—N22—C23	171.9 (3)	F11A—B1—F13A—F13	−105 (4)
C22—C23—N22—C21	20.0 (5)	F12—B1—F13A—F13	129 (4)
C22—C23—N22—C24	−176.3 (4)	F12A—B1—F13A—F13	128 (4)
Cu1—S3—C31—N32	164.1 (3)	F14—B1—F13A—F13	3 (4)
Cu2^i^—S3—C31—N32	−119.9 (3)	F11—B1—F13A—F13	−119 (3)
Cu1—S3—C31—N31	−16.4 (3)	F14A—B1—F13A—F13	15 (4)
Cu2^i^—S3—C31—N31	59.6 (3)	F11A—B1—F14A—F14	−40.6 (18)
N32—C31—N31—C32	−1.5 (4)	F12—B1—F14A—F14	95.5 (16)
S3—C31—N31—C32	178.9 (3)	F13—B1—F14A—F14	−156.2 (16)
N32—C31—N31—S4	−155.7 (3)	F12A—B1—F14A—F14	83 (2)
S3—C31—N31—S4	24.7 (4)	F13A—B1—F14A—F14	−161.8 (16)
C31—N31—C32—C33	1.9 (4)	F11—B1—F14A—F14	−52.1 (19)
S4—N31—C32—C33	157.0 (3)	F21A—B2—F22—F22A	106 (3)
N31—C32—C33—N32	−1.5 (4)	F24—B2—F22—F22A	−8 (3)
N31—C31—N32—C34	178.6 (4)	F21—B2—F22—F22A	111 (3)
S3—C31—N32—C34	−1.8 (6)	F24A—B2—F22—F22A	−27 (3)
N31—C31—N32—C33	0.5 (5)	F23A—B2—F22—F22A	−142 (2)
S3—C31—N32—C33	−180.0 (3)	F23—B2—F22—F22A	−130 (2)
C32—C33—N32—C31	0.8 (5)	F21A—B2—F23—F23A	91 (2)
C32—C33—N32—C34	−177.5 (4)	F24—B2—F23—F23A	−149.7 (19)
C21—N21—S4—N31	101.3 (3)	F21—B2—F23—F23A	91 (2)
C22—N21—S4—N31	−70.6 (3)	F24A—B2—F23—F23A	−156.7 (19)
C21—N21—S4—Cu1	0.3 (3)	F22—B2—F23—F23A	−28 (2)
C22—N21—S4—Cu1	−171.6 (3)	F22A—B2—F23—F23A	−50 (2)
C31—N31—S4—N21	−115.8 (3)	F21A—B2—F24—F24A	106 (3)
C32—N31—S4—N21	92.7 (3)	F21—B2—F24—F24A	101 (3)
C31—N31—S4—Cu1	−15.7 (3)	F22—B2—F24—F24A	−140 (2)
C32—N31—S4—Cu1	−167.3 (3)	F23A—B2—F24—F24A	−34 (3)
Cu2—S5—C51—N52	17.6 (8)	F22A—B2—F24—F24A	−143 (2)
Cu2—S5—C51—N51	−160.5 (5)	F23—B2—F24—F24A	−17 (2)
N52—C51—N51—C52	3.0 (10)	F21A—B2—F22A—F22	−82 (3)
S5—C51—N51—C52	−178.5 (6)	F24—B2—F22A—F22	172 (2)
C51—N51—C52—C53	−6.1 (11)	F21—B2—F22A—F22	−78 (3)
N51—C52—C53—N52	6.5 (12)	F24A—B2—F22A—F22	157 (2)
N51—C51—N52—C54	−176.3 (7)	F23A—B2—F22A—F22	40 (3)
S5—C51—N52—C54	5.5 (12)	F23—B2—F22A—F22	60 (3)
N51—C51—N52—C53	1.7 (11)	F21A—B2—F23A—F23	−100 (2)
S5—C51—N52—C53	−176.6 (9)	F24—B2—F23A—F23	39 (2)
C52—C53—N52—C51	−5.4 (13)	F21—B2—F23A—F23	−96.8 (19)
C52—C53—N52—C54	172.7 (8)	F24A—B2—F23A—F23	25 (2)
Cu2—S5—C51A—N52A	−169.1 (11)	F22—B2—F23A—F23	153.6 (18)
Cu2—S5—C51A—N51A	13.0 (12)	F22A—B2—F23A—F23	139.1 (19)
N52A—C51A—N51A—C52A	−6.4 (15)	F21A—B2—F24A—F24	−82 (3)
S5—C51A—N51A—C52A	171.7 (9)	F21—B2—F24A—F24	−88 (2)
C51A—N51A—C52A—C53A	9.5 (19)	F22—B2—F24A—F24	49 (3)
N51A—C52A—C53A—N52A	−9 (2)	F23A—B2—F24A—F24	153 (2)
N51A—C51A—N52A—C54A	−168.7 (13)	F22A—B2—F24A—F24	39 (2)
S5—C51A—N52A—C54A	13 (2)	F23—B2—F24A—F24	164 (2)

Symmetry codes: (i) *x*, −*y*+1/2, *z*−1/2; (ii) *x*, −*y*+1/2, *z*+1/2.

### Hydrogen-bond geometry (Å, º)

**Table:**

*D*—H···*A*	*D*—H	H···*A*	*D*···*A*	*D*—H···*A*
N11—H11*A*···F12	0.88	2.12	2.764 (11)	129
N11—H11*A*···F12*A*	0.88	2.15	2.74 (2)	124
N51—H51*A*···F23	0.88	2.20	2.995 (12)	150
C13—H13*B*···F12^iii^	0.99	2.63	3.329 (17)	128
C13—H13*B*···F12*A*^iii^	0.99	2.59	3.22 (3)	122
C14—H14*B*···F14*A*^iii^	0.98	2.59	3.331 (14)	133
C14—H14*C*···F11*A*^iv^	0.98	2.64	3.119 (17)	111
C22—H22*B*···F24^v^	0.99	2.63	3.293 (8)	125
C22—H22*B*···F24*A*^v^	0.99	2.61	3.395 (17)	136
C23—H23*A*···F24^v^	0.99	2.56	3.164 (8)	119
C23—H23*B*···F22^vi^	0.99	2.63	3.453 (8)	140
C24—H24*C*···F13	0.98	2.58	3.321 (11)	133
C32—H32*A*···F21	0.99	2.33	3.259 (15)	155
C32—H32*A*···F21*A*	0.99	2.46	3.38 (3)	155
C32—H32*A*···F23*A*	0.99	2.61	3.287 (16)	126
C32—H32*B*···F24^i^	0.99	2.56	3.499 (9)	158
C32—H32*B*···F24*A*^i^	0.99	2.59	3.569 (18)	170
C33—H33*A*···F21^i^	0.99	2.33	3.182 (12)	144
C33—H33*A*···F21*A*^i^	0.99	2.31	3.16 (3)	145
C34—H34*A*···F13*A*^i^	0.98	2.62	3.154 (18)	114
C34—H34*B*···F22^vii^	0.98	2.60	3.247 (8)	123
C53—H53*B*···F13^viii^	0.99	2.36	3.269 (16)	152
C54—H54*A*···S1	0.98	2.89	3.835 (8)	162
C54—H54*C*···F11^i^	0.98	2.39	3.335 (10)	162
C53*A*—H53*D*···F22^vii^	0.99	2.52	3.49 (3)	167
C54*A*—H54*D*···F23	0.98	2.09	2.933 (16)	144
C54*A*—H54*D*···F23*A*	0.98	2.60	3.43 (2)	143
C54*A*—H54*E*···F23*A*^vii^	0.98	2.47	3.36 (2)	152

Symmetry codes: (i) *x*, −*y*+1/2, *z*−1/2; (iii) −*x*, −*y*+1, −*z*+1; (iv) −*x*, *y*−1/2, −*z*+3/2; (v) −*x*+1, *y*+1/2, −*z*+3/2; (vi) −*x*+1, −*y*+1, −*z*+1; (vii) −*x*+1, −*y*, −*z*+1; (viii) *x*, *y*−1, *z*.
